# CBCT Evaluation of Buccolingual Orientation of Inferior Alveolar Canal in Mandibular Posterior Region for Implant Planning

**DOI:** 10.1155/2022/4682105

**Published:** 2022-08-27

**Authors:** Michelle Kher Wei Chua, Wen Jiong Koh, Smita Nimbalkar, Pravinkumar G. Patil

**Affiliations:** ^1^School of Dentistry, International Medical University, Kuala Lumpur, Malaysia; ^2^Division of Clinical Oral Health Sciences, School of Dentistry, International Medical University, Kuala Lumpur, Malaysia; ^3^Division of Restorative Dentistry, School of Dentistry, International Medical University, Kuala Lumpur, Malaysia

## Abstract

**Purpose:**

Inferior alveolar nerve (IAN) can be subjected to iatrogenic injury during implant surgical procedures. The purpose of this retrospective study was to identify the buccolingual orientation of IAN in posterior mandible as adjunct information for implant planning and to estimate ethnicity-, sex-, and side-related variations in Malaysian population. *Material and Methods*. A total of 121 CBCT images were viewed with eXamVision software. The buccolingual position of IAN was identified in the posterior region. Buccal bone width (B), canal thickness (C), and lingual bone width (L) were measured at the horizontal canal levels. Kruskal–Wallis H test and Friedman test were used to analyze the buccolingual position. One-way ANOVA was performed to evaluate the variations in B, C, and L values.

**Results:**

Overall, most of the IANs were located on the lingual sides of the second molar regions (left: 71.9%; right: 71.1%) and at the centers of the first molar regions (left: 57.9%; right: 47.10%) and exited through the mental foramen before the second premolar regions. There was statistically significant difference in the buccolingual position of the IAN between the sexes in the left second premolar regions (*P* = 0.03). There was variation in B between the sexes in the left first molar regions (*P* = 0.01). Statistically significant differences in C and L were also found between different ethnic groups (*P* = 0.04). Between both sides, there were variations in C in the first molar regions (*P* < 0.001) and the second molar regions (*P* = 0.03).

**Conclusion:**

From the second molar to the second premolar, the buccal bone width decreased while the lingual bone width increased. There were variations between ethnicities, sexes, and sides among Malaysians.

## 1. Introduction

The inferior alveolar nerve (IAN) is the branch of the mandibular division of trigeminal nerve that provides innervation to the mandible and the lower teeth. It can be subjected to iatrogenic injury during surgical procedures such as third molar surgery, implant surgery, orthognathic surgery, surgery for pathology, and endodontic therapy. It is interesting to know that the IAN is one of the most commonly injured nerves (64.4%), followed by the lingual nerve (28.8%) [1]. Moreover, up to 40% of incidences are implant-related IAN injuries [1–6]. Intrusion into the inferior alveolar canal during implant placement may result in paraesthesia or complete anesthesia, increased risk of hemorrhage, and impairment of normal functions, such as speech and eating [2]. Hence, the location and course of the IAN are important landmarks in any surgical procedures involving the mandible. The best way to prevent this damage is to have clear three-dimensional (3D) vision of the jaw [2]. This can be achieved by combining the practical knowledge of basic mandibular anatomy and the data obtained from clinical and radiological examination.

Juodzbalys et al. [7] reviewed 46 literature sources concerning the morphological aspects and anatomy variations related to mandibular canal and inferior alveolar neurovascular bundle in implant surgery. They found that in 70% of the cases, the mandibular canal and inferior alveolar neurovascular bundle stretched throughout the mandible body forming an “S” shape curve. Individual, sex, race, assessing technique used, and degree of edentulous alveolar bone atrophy largely influence these variations. It was suggested that implant osteotomies should not be developed in the posterior mandible until the position of the mandibular canal is established. Al-Siweedi et al. [8] studied the anatomical landmarks that can be used to gain access to the inferior alveolar neurovascular bundle. Apicobasal assessment of the canal revealed that it is curving downward toward the inferior mandibular border until 20 mm distal to the mental foramen where it then curves upward, making an elliptic-arc curve. The IAN canal also forms a buccolingually oriented elliptic arc in relation to the buccal cortex.

Cone-beam computed tomography (CBCT) and computed tomography (CT) were used widely for locating the canals and bone thickness. Some literature used CT [9, 10] images while most used CBCT [11–13]. Wang et al. [9] studied the significance of location of mandibular canal using CT in mandibular angle osteotomy surgeries. They measured distances between upper points of lower teeth to the inferior border of the canal and designed the osteotomy according to the canal position to avoid the IAN bundle injury. The mandibular canal was protected very well in all cases (30) without any injury to the IAN bundle. Hsu et al. [11] studied the location of the IAN and the thickness of the occlusal cortical bone at dental implant sites in lower second premolar and lower first molar region using CBCT. The experimental results showed no statistical difference in the distance from the IAN to the upper border of the mandible between the first molar and second premolar. Mirbeigi et al. [12] investigated the buccolingual course of the IAN in different mental foramen locations using CBCT in Iranian population and found that the position of IAN was affected by the location of the mental foramen. The direction of IAN gradually changed from lingual to buccal and from posterior to anterior. Balaji et al. [13] documented a clinically relevant position of the IAN in complete dentate South Indian population aged 20–29 years using CBCT. On average, the lingual cortical thickness was 1.68 mm at first molar and 1.44 mm at second molar level. Pyun et al. [14] investigated the horizontal course of the IAN canal by determining the location of the mental foramen on panoramic radiographs. They found that panoramic radiographs do not provide information on the buccolingual position of the IAN, and the use of computed radiographic technology such as CT or CBCT is greatly advisable.

The purpose of the present study was to find out the buccolingual orientation of the IAN in mandibular premolar and molar regions in Malaysian population (which mainly consists of Malay, Malaysian Chinese, and Malaysian Indian population) and the variations between ethnicities, sexes, and sides. To the authors' knowledge, no such information is available in the literature in Malaysian population.

## 2. Materials and Methods

Institutional ethics committee approval for the study (Approval number: BDS I-01/14 (02)2017) and informed consent from the patients were obtained prior to the study. A total of 121 CBCT Digital Imaging and Communications in Medicine (DICOM) files taken for diagnostic purposes were selected, and 242 hemimandible samples were collected. Following are the inclusion criteria: dentate or edentulous Malay, Malaysian Chinese, or Malaysian Indian patients in the age range from 18 to 80; healthy and medically compromised patients or even those who previously received radiation but not involving the mandible. Exclusion criteria are as follows: patients with mixed racial origins; history of trauma, pathology, or surgical intervention like orthognathic surgery or chin bone harvesting procedures in the mandible; syndromes and congenital disorders; existing pathological disorders of the mandible, such as cysts and tumors, osteomyelitis, and fibrous dysplasia; and distorted or blurred CBCT images due to patients' movements. To achieve a sample covering the three ethnic groups in Malaysia, a stratified random sampling was used to select the CBCT images from each ethnic group (Malay, Malaysian Chinese, Malaysian Indian) by using their clinic registration numbers. A total of 121 participants, namely, 40 Malay patients, 41 Malaysian Chinese patients, and 40 Malaysian Indian patients, were selected for the study. Among them, 61 were male while 60 were female.

The anatomy of the whole mandible was assessed first in the axial, coronal cross-sectional, and panoramic views. The IAN was traced from its entry at the lingula to the mental foramen with the help of eXamVision software (KaVo, Germany) and was highlighted in pink. Two types of parameters were measured: buccolingual position of IAN; the buccal bone width (B), canal thickness (C), and lingual bone width (L). Measurements of these parameters were done on the cross-sectional views. The mesiodistal centers of the premolars and molars were identified to select the cross section for the measurements. In case of edentulous space of some or all the teeth mentioned, the measurements were done in the estimated teeth region.

**Figure 1:**
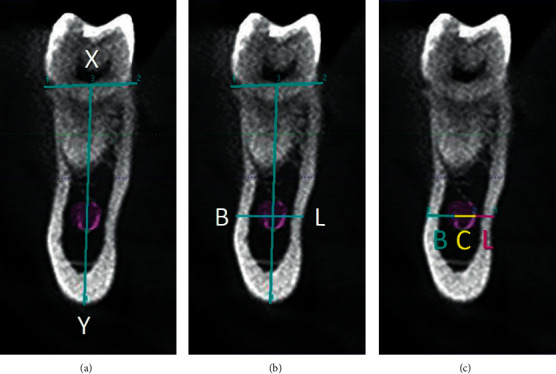
(a) Buccolingual position was determined by line XY; (b) line BL was plotted perpendicular to line XY; (c) buccal bone width (B), canal thickness (C), and lingual bone width (L) were measured along line BL at canal level.

At each cross-sectional view, a horizontal line was drawn along the crest. A line of intersection was drawn at the midpoint of the horizontal line (point X) to lowermost point of the border of the mandible (point Y) (Figure 1(a)). The buccolingual positioning of IAN was determined based on its location as buccal or lingual to the line “XY.” The IAN was considered to be in a buccal (or lingual) position if two-thirds or more of the canal is buccal (or lingual) to the line “XY.”

A “BL” line was then plotted at canal level perpendicular to the line “XY” from buccal to lingual cortex to obtain the values of buccal bone width (B), canal diameter (C), and lingual bone width (L) (Figures 1(b) and 1(c)). The distance B represented the distance between the buccal border of the IAN and the buccal border of the mandible bone; C represented the diameter of the IAN; and L represented the distance between the lingual border of the IAN and the lingual border of the mandible bone. These measurements were recorded on both sides of the mandible.

All data were processed with software (SPSS Statistics v24.0; IBM Corp.). To ensure reliability in the readings, all images were scored by the same observer (MCKW), with 12 randomly selected samples reviewed again 2 weeks later. Cronbach alpha test was calculated to evaluate the reliability between the first and second readings of the selected images. For buccolingual positioning of IAN, Kruskal–Wallis H test was carried out to evaluate the ethnicity- and sex-related variations while Friedman test was performed to determine the side-related variations (*P* ≤ 0.05). In terms of variations in mean values of B, C, and L, one-way analysis of variance (ANOVA) was carried out to evaluate ethnicity-related variations, independent samples *t*-test was performed to determine sex-related variations, and paired samples *t*-test was used to determine side-related variations (*P* ≤ 0.05). Two-way ANOVA was also used to determine the ethnicity-sex variations (*P* ≤ 0.05).

## 3. Results

A Cronbach alpha value of 0.983 indicated excellent reliability of the readings for the same researcher at two different points for the selected images. The mean age of the patients was 46.9 (standard deviation: 17.3). 98.8% of IANs (239 out of 242 hemimandibles) exited through the mental foramen before the first premolar regions. Hence, the location and parameter measurements of IANs in the first premolar regions were omitted.

The buccolingual position of IAN was determined in both sides of the second premolars and the first and second molars (Table 1). Regardless of ethnicity, sex, and side, similar pattern was found in the buccolingual position of IAN. The IANs were located on the lingual sides of the second molar regions, on both right (71.9%) and left sides (71.1%), and at the centers of the first molar regions, on both right (57.9%) and left sides (47.10%), and exited through mental foramen before the second premolar regions. Kruskal–Wallis H test revealed no statistically significant difference in buccolingual positioning of IAN among the three ethnic groups (*P* > 0.05), but there was sex-related variation in the region of right second premolar, 45 (*P*=0.03) (Table 1). Moreover, Friedman test showed that the difference in the buccolingual position of IAN between both sides of all the teeth regions was statistically insignificant (*P* > 0.05) (Table 2).

The mean values of B, C, and L were measured on both sides of the mandible (Table 3). B was the thickest at second molar regions (right, left: 6.13 mm) and the thinnest at second premolar regions (right: 3.10 mm; left: 3.14 mm) while L was the thickest at second premolar regions (right: 4.09 mm; left: 4.36 mm) and the thinnest at second molar regions (right: 2.49 mm; left: 2.58 mm). Overall, B decreased while L increased from second molars to second premolars regardless of ethnicity, sex, and side. C generally increased from second molar regions to second premolar regions on both sides.

One-way ANOVA showed that there were ethnicity-related variations (Table 3) which were further analyzed with Tukey HSD post hoc test to locate the exact differences between ethnic groups (Table 4). Between Malay and Malaysian Chinese patients, there were statistically significant differences in 47C (C value of tooth number 47) (*P* < 0.001), 46C (*P*=0.001), and 37C (*P*=0.02) with Malaysian Chinese group having greater values. Between Malaysian Chinese and Malaysian Indian patients, statistically significant differences in 47C (*P* < 0.001), 47L (*P*=0.01), 37L (*P*=0.001), and 36L (*P* < 0.001) were noted. Malaysian Chinese subjects also presented with greater mean values of the above-mentioned measurements as compared to Malaysian Indian ones. Malay group had greater values in 37L (*P*=0.04) and 36L (*P*=0.02), being statistically significant, than Malaysian Indian group. By contrast, there was no statistically significant difference between the ethnicities in the B values of all the molars and all the measurements (B, C, and L) of the second premolars (*P* > 0.05).

There was no sex-related variation in any molar regions except for 46B (*P*=0.01) (Table 3). Males had thicker buccal bone width in the region 46 (4.77 mm) as compared to females (4.15 mm). Between both sides, there were variations in C of first molars (*P* < 0.001) and second molars (*P*=0.03) with greater thickness presented on the right side (Table 2). In terms of variations between ethnicities and sexes, the findings in second premolars were omitted due to high absence rate of IAN in this region. The ethnicity-sex differences in the mean values of B, C, and L in molar regions were not statistically significant (*P* > 0.05) (Table 5).

## 4. Discussion

Inferior alveolar nerve (IAN) injury can occur in any phase of dental implant surgery, including local anesthetic administration, incision, soft tissue reflection, implant osteotomy, implant placement, and post-surgery suturing. It is important to have in-depth knowledge about the location and course of IAN canal before any surgery procedure to minimize the risk of IAN injury. Various imaging modalities, such as periapical radiograph, panoramic radiograph, CT, and CBCT have been used to pre-assess the sites of surgery like implant sites. CBCT was used for this study as it provides clear and accurate images of structures and is useful for assessing the bony component. It overcomes the inherent limitation of the periapical and panoramic radiograph. Periapical and panoramic radiograph are two dimensional views of 3D anatomic landmarks and can cause geometric distortion, superimposition, and alteration of vertical dimension [15]. Moreover, the mandibular canal presented with better visibility on CBCT images than on panoramic radiograph and caused lower radiation exposure as compared with the conventional CT [16–18].

The buccolingual course of IAN in the present study appeared to follow the findings from the studies by Juodzbalys et al. [7], Al-Siweedi et al. [8], Mirbeigi et al. [12], and Pyun et al. [14] where most of the nerves stretched from the lingual sides of the second molar regions to the centers of the first molar regions and exited before the second premolar regions through mental foramen. Juodzbalys et al. [7] suggested that individual, gender, age, race, assessing technique used, and degree of edentulous alveolar bone atrophy largely influenced these variations. The present study suggested that there were sex-related variations in buccolingual position of IANs in right second premolar regions. Most of the IANs exited through mental foramen in second premolar regions, but in subjects where IANs were still found, males presented with higher percentage of IANs located at the center while females showed higher percentage of IANs located on the buccal side. The variation between sexes is due to difference in the degree of bone growth in the adult phase which is controlled by sex hormones and muscular tension [19]. Sex hormones such as estrogen and progesterone can influence the speed of bone growth, contributing to the differences in the development of the craniofacial morphology between both sexes [19].

In the present study, the mean values of buccal bone width decreased while the lingual bone width increased from the second molar regions to the second premolar regions. This finding is in line with the studies done by Mirbeigi et al. [12] and Balaji et al. [13] which ascribed it to the anatomy of mandible body [12] and consistent jaw remodeling due to the oral musculature attachments such as mylohyoid and masseter muscles [13]. Overall, mean values of the IAN canal thickness decreased from premolars to molars due to the decrease in thickness of vessels which is consistent with Balaji et al. [13] and Humphries [20] studies.

In the current study, there was no variation in buccolingual position of IAN between both sides. However, the differences in canal thickness at first and second molar regions were statistically significant (*P* < 0.001, *P*=0.03). The canal thickness in first and second molar regions were greater on the right side (3.19 mm, 3.00 mm) as compared to the left side (2.89 mm, 2.84 mm), indicating that the right and left halves of mandible are not entirely symmetrical, which is in agreement with Balaji et al. [13] study.

Besides, this study showed that there were no variations in the mean values of B, C, and L between both sexes except for the buccal bone width in right first molar regions (*P*=0.01). Males (4.77 mm) had thicker buccal bone width in the right first molar regions compared to females (4.15 mm). This finding was in line with a study done by Al-Siweedi et al. [8] who stated that the gender influence was only noted for one measurement on the buccal surface of the mandible; the measurement for males (6.09 ± 1.70 mm) was significantly higher than that for females (5.28 ± 1.47 mm) at 3 cm distal to the mental foramen. These findings were also consistent with the studies done by Rath et al. [21] in Eastern India, Gopal and Sundaram [22] in India, and Gamba et al. [23] in Brazil. All three studies showed that there was statistical significance in the distance from the most buccal point of IAN to mandibular cortical plate but no statistical significance in the distance from the most lingual point of IAN to mandibular cortical plate between sexes. Another study conducted by Angel et al. [24] found that most of the measured distances showed no significant difference between sexes in American population, which is in line with this study. The findings in this study were slightly not in accordance with the studies done by Mirbeigi et al. [12], Balaji et al. [13], Mousa et al. [19], and Nagadia et al. [25] where all the buccal and lingual distances were not statistically significant between both sexes. This difference may be caused by the various ethnicities of patients used in each study as Mirbeigi et al. [12] studied Iranian population, Balaji et al. [13] studied South Indian population, and Mousa et al. [19] studied Egyptian population while Nagadia et al. [25] used Chinese population as their subject samples. These studies examined different populations, and the variations in genetics, diet, habits, and customs may result in distinct anatomic features [26].

In the current study, there was no ethnic-related variation in buccal bone width, but there were variations between ethnicities in IAN canal thickness and lingual bone width. By contrast, Levine et al. [10] suggested that race was statistically associated with IAN position relative to the buccal cortical mandibular margin. This difference may be due to sampling from different races as well. The patients in the current study were specifically Malaysian population involving Malay, Malaysia Chinese, and Malaysian Indian groups while in Levine's study the participants were American involving white and black races. From the current study, Malaysian Chinese participants generally had the greatest values of B, C, and L while Malaysian Indian participants had the least ones, indicating that Malaysian Chinese group had the largest buccolingual distance of posterior mandible, followed by Malay and Malaysian Indian groups. This finding was similar to that of Al-Siweedi et al. [8] who suggested that Chinese subjects in general presented with measurements that were furthest from the buccal cortex of the mandible when compared to other two ethnic groups while Indians presented with the shortest distance between the IAN canal and buccal cortex. There was limited literature comparing the buccal bone width, canal thickness, and lingual bone width among the three ethnicities in Malaysian population.

Understanding the position of IAN and available bone width is crucial in performing a successful implant placement. Failure in achieving this can lead to serious complications, such as neurosensory disturbances, impairment of motor function, severe bleeding, edema, hematoma, and failure due to soft tissue migration. The present study provided detailed information regarding the buccolingual position of IAN and available buccal and lingual bone widths of the IAN among Malaysian population. It serves as references for the safe zone determination in implant planning. By knowing the buccolingual orientation of IAN, the angle of entry and the buccolingual angulation of the implant can be planned in advance, to prevent iatrogenic nerve injury and reduce the risk of complications.

The current study showed that there were ethnicity-, sex-, and side-associated variations in buccolingual orientation of IAN. These mentioned variations should be taken into consideration during surgical procedures involving mandible. Due to these anatomical variations, 3D imaging of the jaw was essential to reduce undesired complications. The CBCT has been widely used in visualizing the position of IAN, the bony component, and the safe zone for implant placement. The IAN is an important landmark during implant placement in posterior mandible to prevent iatrogenic nerve injury. With the knowledge of the buccolingual position of IAN and the available bone width buccal and lingual to IAN, the IAN can be predicted at specific tooth region and can be used as adjunct information prior to implant placement. The implant placement can be planned based on the bone sounding, panoramic radiographs, and this available adjunct information especially when the CT scan facility is not available in the clinical setting.

A study by Ulm et al. [27] has shown that the distance of the mandibular canal to the external lingual and buccal cortical plates remained remarkably constant with increasing atrophy. Thus, the authors believe that the results obtained were consistent, regardless of whether the patients were dentate or edentulous. However, further studies with bigger sample sizes are needed to confirm the suggestion that dentition status does not influence the results obtained.

This study was subjected to some limitations. The dentition status of the samples was not taken into consideration. Edentulous or partially edentulous mandibles have not been considered for the measurements. In future study, the difference of dentition status in the location of IAN can be included. Besides, in edentulous ridges, the tooth area was determined by estimation which may not be accurate.

## 5. Conclusions

Within the limitations of this study, the following conclusions can be drawn:

1. IANs extended from the lingual sides of the second molar regions (71.5%) to the centers of the first molar regions (52.5%) and exited before the second premolar regions (65.7%) through the mental foramen.
2. The mean values of buccal bone width increased from the second premolar regions to the second molar regions while the lingual bone width decreased.
3. As regards the buccolingual positioning of IAN, there were sex-related variations in specific tooth regions.
4. There were ethnicity- and side-related significant variations in the canal thickness, mainly in the molar regions. Variation of the buccal bone width was also noted between sexes while variation of lingual bone width in the molar regions was noted among ethnicity groups.

## Figures and Tables

**Table 1:** The buccolingual positioning of IAN canal in each respective tooth according to the ethnicity and sex group among Malaysian population.

		Location of IAN canal	Ethnic, *n* (%)						*P* value	Sex, *n* (%)				*P* value	Overall (*N* = 121)	
			Malay (*N* = 40)		Chinese (*N* = 41)		Indian (*N* = 40)			Male (*N* = 61)		Female (*N* = 60)				
Right	Second molar (47)	Buccal	0	(0)	1	(2.4)	1	(2.5)	0.16	1	(1.6)	1	(1.7)	0.65	2	(1.7)
		Center	9	(22.5)	15	(36.6)	8	(20.0)		15	(24.6)	17	(28.3)		32	(26.4)
		Lingual	31	(77.5)	25	(61.0)	31	(77.5)		45	(73.8)	42	(70.0)		87	(71.9)
		Absent	0	(0)	0	(0)	0	(0)		0	(0)	0	(0)		0	(0)
	First molar (46)	Buccal	4	(10.0)	7	(17.1)	3	(7.5)	0.86	8	(13.1)	6	(10.0)	0.09	14	(11.6)
		Center	23	(57.5)	21	(51.2)	26	(65.0)		29	(47.5)	41	(68.3)		70	(57.9)
		Lingual	12	(30.0)	12	(29.3)	11	(27.5)		24	(39.3)	11	(18.3)		35	(28.9)
		Absent	1	(2.5)	1	(2.4)	0	(0)		0	(0)	2	(3.3)		2	(1.7)
	Second premolar (45)	Buccal	10	(25.0)	4	(9.8)	7	(17.5)	0.07	8	(13.1)	13	(18.0)	0.03^*∗*^	21	(17.4)
		Center	40	(10.0)	8	(19.5)	7	(17.5)		11	(18.0)	8	(13.3)		19	(15.7)
		Lingual	1	(2.5)	3	(7.3)	0	(0)		4	(6.6)	0	(0)		4	(3.3)
		Absent	25	(62.5)	26	(63.4)	26	(65.0)		38	(62.3)	39	(65.0)		77	(63.6)

Left	Second molar (37)	Buccal	0	(0)	2	(4.9)	20	(5.0)	0.38	1	(1.6)	3	(5.0)	0.26	4	(3.3)
		Center	14	(35.0)	11	(26.8)	6	(15.0)		14	(23.0)	17	(28.3)		31	(25.6)
		Lingual	26	(65.0)	28	(68.3)	32	(80.0)		46	(75.4)	40	(66.7)		86	(71.1)
		Absent	0	(0)	0	(0)	0	(0)		0	(0)	0	(0)		0	(0)
	First molar (36)	Buccal	7	(17.5)	7	(17.1)	7	(17.5)	0.96	9	(14.8)	12	(20.0)	0.40	21	(17.4)
		Center	19	(47.5)	19	(46.3)	19	(47.5)		29	(47.5)	28	(46.7)		57	(47.1)
		Lingual	13	(32.5)	15	(36.6)	14	(35.0)		23	(37.7)	19	(31.7)		42	(34.7)
		Absent	1	(2.5)	0	(0)	0	(0)		0	(0)	1	(1.7)		1	(0.8)
	Second premolar (35)	Buccal	3	(7.5)	4	(9.8)	6	(15.0)	0.65	7	(11.5)	6	(10.0)	0.35	13	(10.7)
		Center	6	(15.0)	3	(7.3)	11	(27.5)		11	(18.0)	9	(15.0)		20	(16.5)
		Lingual	0	(0)	4	(9.8)	2	(5.0)		5	(8.2)	1	(1.7)		6	(5.0)
		Absent	31	(77.5)	30	(73.2)	21	(52.5)		38	(62.3)	44	(73.3)		82	(67.8)

^*∗*^Statistically significant difference (*P* < 0.05).

**Table 2:** Friedman test and paired samples *t*-test results showing the variation in the location of IAN canal and B, C, and L values between sides, respectively.

	Location of IAN canal	B, C, and L	*P* value
	*P* value		
Second molar	0.56	B	0.99
		C	0.03^*∗*^
		L	0.48

First molar	0.88	B	0.81
		C	<0.001^*∗*^
		L	0.21

Second premolar	0.29	B	0.82
		C	0.21
		L	0.90

B: buccal bone width; C: canal thickness; L: lingual bone width of IAN. ^*∗*^Statistically significant difference (*P* < 0.05).

**Table 3:** The one-way ANOVA and independent *t*-test results showing the variations in the mean values of B, C, and L according to the ethnicity and sex group among Malaysian population.

			Ethnicity			*P* value	Sex		*P* value	Overall (*N* = 121)
			Malay (*n* = 40)	Chinese (*n* = 41)	Indian (*n* = 40)		Male (*n* = 61)	Female (*n* = 60)		
Right	Second molar (47)	B	6.23	6.08	6.08	0.89	6.19	6.07	0.06	6.13
		C	2.80	3.48	2.71	<0.001^*∗*^	3.13	2.87	0.70	3.00
		L	2.48	2.92	2.07	0.01^*∗*^	2.54	2.44	0.06	2.49
	First molar (46)	B	4.60	4.71	4.09	0.13	4.77	4.15	0.01^*∗*^	4.46
		C	2.88	3.50	3.19	0.001^*∗*^	3.35	3.03	0.95	3.19
		L	3.30	3.31	2.65	0.04^*∗*^	2.88	3.30	0.37	3.09
	Second premolar (45)	B	3.01	3.47	2.80	0.38	3.33	2.85	0.52	3.10
		C	3.08	3.60	3.22	0.08	3.32	3.28	0.39	3.30
		L	3.78	4.20	4.30	0.68	4.08	4.10	0.60	4.09

Left	Second molar (37)	B	6.29	6.38	5.72	0.15	6.23	6.03	0.73	6.13
		C	2.65	3.07	2.79	0.02^*∗*^	3.05	2.62	0.13	2.84
		L	2.65	2.98	2.09	0.001^*∗*^	2.46	2.70	0.32	2.58
	First molar (36)	B	4.42	4.77	4.21	0.30	4.76	4.17	0.62	4.47
		C	2.84	2.97	2.86	0.58	2.97	2.81	0.10	2.89
		L	3.40	3.74	2.63	0.001^*∗*^	3.14	3.38	0.25	3.26
	Second premolar (35)	B	3.83	3.14	2.82	0.19	3.10	3.21	0.33	3.14
		C	2.89	3.21	2.90	0.50	3.12	2.80	0.66	2.99
		L	4.43	5.01	3.96	0.15	4.38	4.33	0.58	4.36

B: buccal bone width; C: canal thickness; L: lingual bone width of IAN in mm. ^*∗*^Statistically significant difference (*P* < 0.05).

**Table 4:** The post hoc test results showing the mean differences in statistically significant measurements among the three ethnic groups (Malay, Malaysian Chinese, Malaysian Indian).

Location	Ethnic A	Ethnic B	Mean difference (*A* − B)	*P* value
47C	Malay	Chinese	−0.68	<0.001^*∗*^
	Malay	Indian	0.09	0.83
	Chinese	Indian	0.76	<0.001^*∗*^

47L	Malay	Chinese	−0.44	0.27
	Malay	Indian	0.41	0.33
	Chinese	Indian	0.85	0.01^*∗*^

46C	Malay	Chinese	−0.62	0.001^*∗*^
	Malay	Indian	−0.31	0.15
	Chinese	Indian	0.32	0.13

46L	Malay	Chinese	−0.01	1.00
	Malay	Indian	0.64	0.07
	Chinese	Indian	0.66	0.06

37C	Malay	Chinese	−0.41	0.02^*∗*^
	Malay	Indian	−0.14	0.61
	Chinese	Indian	0.27	0.16

37L	Malay	Chinese	−0.33	0.33
	Malay	Indian	0.56	0.04^*∗*^
	Chinese	Indian	0.89	0.001^*∗*^

36L	Malay	Chinese	−0.34	0.47
	Malay	Indian	0.77	0.02^*∗*^
	Chinese	Indian	1.11	<0.001^*∗*^

C: canal thickness; L: lingual bone width of IAN. ^*∗*^Statistically significant difference (*P* < 0.05).

**Table 5:** Two-way ANOVA results showing the ethnicity-sex variations in the mean values of B, C, and L.

			Malay		Chinese		Indian		*P* value
			Male	Female	Male	Female	Male	Female	
Right	Second molar (47)	B	6.24	6.23	6.03	6.19	6.39	5.80	0.58
		C	2.95	2.72	3.55	3.35	2.68	2.75	0.56
		L	2.42	2.52	3.07	2.63	1.90	2.23	0.41
	First molar (46)	B	5.18	4.23	4.88	4.34	4.27	3.93	0.63
		C	3.04	2.78	3.59	3.32	3.25	3.13	0.87
		L	2.90	3.55	3.18	3.59	2.45	2.84	0.89

Left	Second molar (37)	B	6.60	6.10	6.36	6.41	5.74	5.69	0.75
		C	2.68	2.63	3.26	2.68	3.03	2.58	0.17
		L	2.41	2.80	2.93	3.10	1.85	2.31	0.81
	First molar (36)	B	4.75	4.21	4.83	4.64	4.66	3.80	0.67
		C	2.81	2.86	3.04	2.82	2.99	2.73	0.48
		L	3.23	3.51	3.73	3.75	2.24	2.99	0.45

B: buccal bone width; C: canal thickness; L: lingual bone width of IAN in mm.

## Data Availability

The data used to support the findings of this study are available from the corresponding author upon.

## References

[1] Tay A., Zuniga J. (2007). Clinical characteristics of trigeminal nerve injury referrals to a university centre. *International Journal of Oral and Maxillofacial Surgery*.

[2] Juodzbalys G., Wang H. L., Sabalys G. (2011). Injury of the inferior alveolar nerve during implant placement: a literature review. *Journal of Oral & Maxillofacial Research*.

[3] Lin C. S., Wu S. Y., Huang H. Y., Lai Y. L. (2016). Systematic review and meta-analysis on incidence of altered sensation of mandibular implant surgery. *PLoS One*.

[4] Dao T. T., Mellor A. (1998). Sensory disturbances associated with implant surgery. *The International Journal of Prosthodontics*.

[5] Ellies L. G., Freeman K., Kraut R. A. (1999). The incidence of altered sensation of the mental nerve after mandibular implant placement. *Journal of Oral and Maxillofacial Surgery*.

[6] Ellies L. G., Hawker P. B. (1993). The prevalence of altered sensation associated with implant surgery. *The International Journal of Oral & Maxillofacial Implants*.

[7] Juodzbalys G., Wang H. L., Sabalys G. (2010). Anatomy of mandibular vital structures. part I: mandibular canal and inferior alveolar neurovascular bundle in relation with dental implantology. *Journal of Oral & Maxillofacial Research*.

[8] Al-Siweedi S. Y. A., Nambiar P., Shanmuhasuntharam P., Ngeow W. C. (2014). Gaining surgical access for repositioning the inferior alveolar neurovascular bundle. *The Scientific World Journal*.

[9] Wang J. C., Gui L., Zhang Z. Y., Niu F. C. J., Cai J. L. (2008). Significance of location of mandibular canal by 3-dimensional CT in the mandibular angle osteotomy. *Zhonghua Zhengxing Waike Zazhi*.

[10] Levine M. H., Goddard A. L., Dodson T. B. (2007). Inferior alveolar nerve canal position: a clinical and radiographic study. *Journal of Oral and Maxillofacial Surgery*.

[11] Hsu J. T., Huang H. L., Fuh L. J. (2013). Location of the mandibular canal and thickness of the occlusal cortical bone at dental implant sites in the lower second premolar and first molar. *Computational and Mathematical Methods in Medicine*.

[12] Mirbeigi S., Safaee A., Ezoddini F., Khojastepour L., Navab-Azam A. (2016). Buccolingual course of the inferior alveolar canal in different mental foramen locations: a cone beam computed tomography study of an Iranian population. *International Journal of Applied and Basic Medical Research*.

[13] Balaji S. M., Krishnaswamy N., Kumar S., Rooban T. (2012). Inferior alveolar nerve canal position among south Indians: a cone beam computed tomographic pilot study. *Ann Maxillofac Surg*.

[14] Pyun J. H., Lim Y. J., Kim M. J., Ahn S. J., Kim J. (2012). Position of the mental foramen on panoramic radiographs and its relation to the horizontal course of the mandibular canal: a computed tomographic analysis. *Clinical Oral Implants Research*.

[15] Razi T., Moslemzade S. H., Razi S. (2009). Comparison of linear dimensions and angular measurements on panoramic images taken with two machines. *Journal of Dental Research, Dental Clinics, Dental Prospects*.

[16] Angelopoulos C., Thomas S., Hechler S., Parissis N., Hlavacek M. (2008). Comparison between digital panoramic radiography and cone-beam computed tomography for the identification of the mandibular canal as part of presurgical dental implant assessment. *Journal of Oral and Maxillofacial Surgery*.

[17] Jung Y. H., Cho B. H. (2014). Radiographic evaluation of the course and visibility of the mandibular canal. *Imaging Sci Dent*.

[18] Ludlow J. B., Ivanovic M. (2008). Comparative dosimetry of dental CBCT devices and 64-slice CT for oral and maxillofacial radiology. *Oral Surgery, Oral Medicine, Oral Pathology, Oral Radiology & Endodontics*.

[19] Mousa A., El Dessouky S., El Beshlawy D. (2020). Sex determination by radiographic localization of the inferior alveolar canal using cone-beam computed tomography in an Egyptian population. *Imaging Science in Dentistry*.

[20] Humphries S. (2007). Comparison of cortical bone thickness between second premolars and first molars in the maxilla and mandible in four ethnic groups.

[21] Rath R., Sangamesh N. C., Acharya R. R., Sharma G. (2022). Sexual dimorphism of inferior alveolar canal location: a record-based CBCT study in eastern India. *Journal of Oral and Maxillofacial Pathology*.

[22] Gopal S., Sundaram S. (2017). Sexual dimorphism by locating the mandibular canal in different positions using images from cone beam computed tomography. *American Journal of Oral Medicine and Radiology*.

[23] Gamba T. d O., Alves M. C., Haiter-Neto F. (2014). Analysis of sexual dimorphism by locating the mandibular canal in images of cone-beam computed tomography. *Journal of Forensic Radiology and Imaging*.

[24] Angel J. S., Mincer H. H., Chaudhry J., Scarbecz M. (2011). Cone-beam computed tomography for analyzing variations in inferior alveolar canal location in adults in relation to age and sex. *Journal of Forensic Sciences*.

[25] Nagadia R., Tay A. B. G., Chan L. L., Chan E. S. Y. (2011). The spatial location of the mandibular canal in Chinese: a CT study. *International Journal of Oral and Maxillofacial Surgery*.

[26] Uysal T., Yagci A., Aldrees A. M., Ekizer E. (2011). Ethnic differences in dentofacial relationships of Turkish and Saudi young adults with normal occlusions and well-balanced faces. *The Saudi Dental Journal*.

[27] Ulm C., Solar P., Blahout R. (1993). Location of the mandibular canal within the atrophic mandible. *British Journal of Oral and Maxillofacial Surgery*.

